# The Aging Heart: Mitophagy at the Center of Rejuvenation

**DOI:** 10.3389/fcvm.2020.00018

**Published:** 2020-02-19

**Authors:** Wenjing J. Liang, Åsa B. Gustafsson

**Affiliations:** Department of Pharmacology, Department of Medicine, Skaggs School of Pharmacy and Pharmaceutical Sciences, University of California, San Diego, CA, United States

**Keywords:** aging, autophagy, mitophagy, mitochondria, heart, PINK1, Parkin, mitophagy receptors

## Abstract

Aging is associated with structural and functional changes in the heart and is a major risk factor in developing cardiovascular disease. Many recent studies have focused on increasing our understanding of the basis of aging at the cellular and molecular levels in various tissues, including the heart. It is known that there is an age-related decline in cellular quality control pathways such as autophagy and mitophagy, which leads to accumulation of potentially harmful cellular components in cardiac myocytes. There is evidence that diminished autophagy and mitophagy accelerate the aging process, while enhancement preserves cardiac homeostasis and extends life span. Here, we review the current knowledge of autophagy and mitophagy in aging and discuss how age-associated alterations in these processes contribute to cardiac aging and age-related cardiovascular diseases.

## Introduction

Aging is a major risk factor in developing cardiovascular disease and increases exponentially with age. Cardiac aging is characterized by the presence of hypertrophy, fibrosis, accumulation of misfolded proteins, and dysfunctional mitochondria. Current efforts are dedicated to understanding the biological process of aging and to identify pathways that can be targeted to extend health and life spans. Interestingly, it has been demonstrated that many of the pathways that improve health and extend longevity in various organisms all converge on autophagy (1–8). Autophagy is a catabolic pathway that is responsible for recycling cellular proteins and organelles to maintain energy homeostasis. It participates in the elimination of pathogens and prevents activation of inflammation. It is also a key pathway in cellular quality control by eliminating dysfunctional or unwanted organelles and protein aggregates. However, there is strong evidence that autophagy is decreased with age in tissues, including the heart (5, 9–15).

The heart requires a lot of energy which is mainly generated by mitochondria via oxidative phosphorylation. However, aging is associated with altered cardiac mitochondrial metabolism and mitochondrial respiratory defects (16). The impaired fatty acid and glucose metabolism, combined with reduced mitochondrial respiration are also believed to underlie the increased susceptibility to cardiac injury in the elderly population (16). Normally, these dysfunctional mitochondria are eliminated by autophagosomes in a selective process termed mitophagy. Predictably, reduced autophagy in aging contributes to accumulation of dysfunctional mitochondria and decreased ability to adapt to stress.

Altered autophagy and mitophagy overtime are likely central contributors in the aging process. Here, we review the current knowledge of autophagy and mitophagy in aging and discuss how age-associated alterations in these processes contribute to cardiac aging and age-related cardiovascular diseases.

## Autophagy

**Figure 1:**
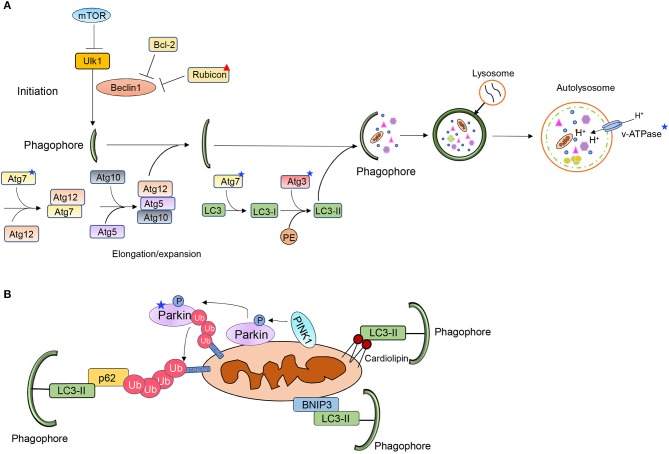
Overview of **(A)** autophagy and **(B)** mitophagy pathways. Stars and triangle mark proteins that have been reported to be altered with age.

Autophagy involves the sequestration of ubiquitinated cargo into vesicles called autophagosomes and delivery of the content to lysosomes via fusion. The cargo is degraded inside lysosomes and the components are recycled to the cytoplasm. Autophagy is a highly regulated process and consists of several distinct steps; initiation, nucleation and formation of phagophore, sequestration of cargo, and fusion of autophagosome with a lysosome (Figure 1A). The different steps in the process are regulated by different autophagy-related proteins (Atg) (17). The mechanistic target of rapamycin (mTOR) functions as a gate keeper and prevents activation of autophagy. When mTOR is inhibited, it leads to activation of the unc-51 like autophagy activating kinase 1 (Ulk1/Atg1) which initiates the nucleation of the autophagosome via Beclin1 (18). At baseline, Beclin1 is sequestered by Bcl-2 and Rubicon to suppress autophagy but its release allows it to initiate autophagosome formation (19–21). The elongation and maturation of the growing autophagosome membrane requires two conjugation pathways. The E1-like and E2-like enzymes Atg7 and Atg10 conjugate Atg5 to Atg12. The Atg5-Atg12 complex then interacts with Atg16. Atg16 is required for the proper localization of the complex to the pre-autophagosomal membrane (22). The Atg5-12-16 complex then functions as an E3-like enzyme in the second conjugation pathway, where LC3 is covalently linked to phosphatidylethanolamine (PE). The conjugation of LC3 to PE to form LC3II is mediated by Atg7 (E1-like) and Atg3 (E2-like), respectively (17). LC3II is also involved in cargo recognition where it binds to adaptor proteins such as p62 (23). Several proteins in this pathway are altered with age which ultimately leads to diminished autophagy.

## Mitophagy

### PINK1/Parkin-Mediated Mitophagy

The PINK1/Parkin pathway contains three key elements: a mitochondrial membrane depolarization sensor (PINK1), a signal amplifier (Parkin) and a downstream signal effector (ubiquitin chains) (Figure 1B) (24). Under normal cellular conditions, PINK1 is partly imported into the inter mitochondrial membrane space where it is cleaved by resident proteases such as the presenilin-associated rhomboid-like protease (PARL) (25, 26). However, this process is disrupted upon loss of mitochondrial membrane potential, leading to accumulation of PINK1 on the outer mitochondrial membrane (OMM), where PINK1 in turn recruits the E3 ubiquitin ligase Parkin (25, 27, 28). PINK1 phosphorylates both ubiquitin and Parkin which contribute to both its activation and anchoring at the mitochondria (29). PINK1 has also been reported to phosphorylate MFN2 which then functions as a docking site for Parkin at mitochondria (30). This allows activated Parkin to ubiquitinate various outer mitochondrial membrane proteins (31). However, a recent study reported an alternative function for MFN2 during mitophagy where MFN2 must be degraded for mitophagy to proceed (32). MFN2 is known to tether mitochondria to ER at specific contact sites. McLelland et al. found that Parkin-mediated ubiquitination and degradation of MFN2 disrupts the contact sites and releases mitochondria from the ER. The release provides Parkin full access to its other substrates and allows for mitophagy to proceed (32). The mitochondrial proteins ubiquitinated by Parkin are recognized by various adaptor proteins, such as p62/SQSTM1 and Optineurin (33, 34). These adaptors bind to the ubiquitin-chains on proteins in the OMM via their ubiquitin-associated (UBA) domain and simultaneously directly interact with LC3 on the autophagosome via their LC3 Interacting Region (LIR) motifs (23, 33, 35).

### Mitophagy Receptors

Mitochondrial proteins in the OMM can also target mitochondria to autophagosomes (Figure 1B). BNIP3, NIX/BNIP3L, FUNDC1, Bcl2L13, FKBP8, and Prohibitin-2 (PHB2) are some of the mitophagy receptors that have been identified to date (36–41). These proteins are integrated mitochondrial membrane proteins that are facing the cytosol. The exception is PHB2, which is localized in the inner mitochondrial membrane. PHB2 promotes removal of remaining mitochondrion after outer membrane rupture (36). The mitophagy receptors contain LIRs and can therefore bind directly to LC3 on the autophagosome membrane bypassing the need for ubiquitin and adaptor proteins. The phospholipid cardiolipin can also function as a mitophagy receptor (Figure 1B). Cardiolipin is localized on the inner mitochondrial membrane but is externalized on dysfunctional mitochondria where it facilitates mitophagy by interacting with LC3 (42). However, it is possible that, similar to PHB2, cardiolipin can ensure mitophagy of the inner mitochondrial compartment after outer mitochondrial membrane rupture. Although they have all been established as mitophagy receptors, it is unclear how most of them are activated to induce mitophagy of mitochondria. These proteins are also known to have alternative functions and how they switch between the two functions is not completely clear.

The physiological conditions dictating activation of the two distinct mitophagy pathways are still unclear and under intense investigation. Recently, it has been proposed that PINK1/Parkin-mediated mitophagy plays a minimal role in basal mitophagy (43, 44) and that this pathway plays a more important role in stress adaptation and repair (45, 46). Other studies have reported that mitophagy receptors are key regulators of programmed mitophagy during development or differentiation (47–49). Thus, the two different mitophagy pathways appear to have distinct functions in the cell but additional studies are clearly needed. Moreover, cross talk clearly exists between the two mitophagy pathways (50, 51). For instance, the protein phosphatase PGAM5 dephosphorylates FUNDC1 which enhances the interaction between FUNDC1 and LC3 (52). PGAM5 also coordinates with PHB2 to promote PINK1/Parkin-mediated mitophagy where PHB2 decreases PINK1 processing by inhibiting PARL while PGAM5 stabilizes PINK1 on the OMM (53). Taken together, there is clearly coordination between these two pathways, and they can compensate for each other to some extent.

## Autophagy and Aging

A growing body of data support the anti-aging effects of enhanced autophagy. Many studies have demonstrated that enhancing autophagy by limiting caloric intake, genetic manipulation or pharmacological treatments increases lifespan in various organisms (1–6). For instance, transgenic mice with systemic overexpression of Atg5 have enhanced autophagic activity in tissues which leads to health benefits such as reduced weight gain with age and extended life spans compared to wild type mice (2). Although this study did not specifically focus on the myocardium, the authors reported increased autophagic activity as well as reduced fibrosis with age in hearts of the transgenic mice. The cardioprotective effects of enhanced autophagy during the aging process were recently confirmed by the Levine group, who developed a *Becn1*^*F*121*A*/*F*121*A*^ knock-in mouse model with constitutively increased basal autophagy due to a disruption in the Bcl-2 binding to Beclin1. They found that health and life spans are significantly increased in the knock-in mice. Moreover, aged *Becn1*^*F*121*A*/*F*121*A*^ knock-in mice have reduced cardiac hypertrophy and interstitial fibrosis compared to aged-matched wild type mice (20), confirming that preserving autophagy in the heart delays or even prevents cardiac aging. In contrast, selective disruption of autophagy in the heart leads to accelerated cardiac aging with accumulation of ubiquitinated proteins and dysfunctional mitochondria and development of cardiac hypertrophy (54). Preserving autophagy is clearly critical in the heart to prevent biological aging.

## Mitophagy and Aging

Reduced mitophagy also recapitulates the age-related accumulation of dysfunctional mitochondria in tissues. Thus, the forced increase in autophagy in the above studies can also be linked to enhanced mitophagy as it would enhance elimination of dysfunctional mitochondria. Several studies have confirmed that genetic and pharmacological interventions promoting enhanced mitophagy also lead to extended life span (55, 56), while disrupting mitophagy leads to accelerated aging phenotypes (57, 58). For instance, Urolithin A is a natural compound that induces mitophagy and extends life span in *C.elegans* (56). Both systemic and neuron-specific overexpression of Parkin in flies slows aging and extends lifespan, although lifespan extension is greater with ubiquitous Parkin overexpression (59). A link also exists between Parkin-mediated mitophagy and NLRP3 inflammasome activation. The NLRP3 inflammasome is activated by the presence of mitochondrial DNA in the cytosol that have been released from damaged mitochondria. Thus, Parkin-mediated mitophagy of damaged mitochondria functions to prevent activation of the inflammasome (60). The PINK1/Parkin pathway also diminishes STING-induced inflammation by a similar mechanism (61).

Several early studies reported that PINK1 or Parkin deficiency in Drosophila causes accumulation of dysfunctional mitochondria, flight muscle degeneration and reduced lifespan (62–64). Also, Cornelissen et al. found that mitophagic activity in flight muscle increased with aging in flies and that the age-dependent rise is abrogated by either PINK1 or Parkin deficiency (57). Parkin-deficient mice have an accelerated aging phenotype and accumulate aberrant mitochondria in aging heart (58, 65) while cardiac specific overexpression of Parkin can delay cardiac aging by enhancing mitochondrial turnover (65). These studies present evidence that enhancing mitophagy by targeting the Parkin pathway is beneficial. However, the anti-aging effect of Parkin is likely dose-dependent as aged transgenic mice with higher levels of Parkin overexpression develop cardiac fibrosis likely due to an imbalance between ubiquitination and autophagic degradation (66).

Much less is known about what happens to mitophagy receptors during aging. It was recently reported that mice deficient in both Akt2 and AMPK are predisposed to cardiac aging possible due to compromised mitophagy. These hearts have reduced levels of several mitophagy proteins including BNIP3 and FUNDC1 (15). A mouse model carrying a proofreading-defective mtDNA polymerase γ (POLG) accumulate mtDNA mutations which leads to accelerated aging (67). Unexpectedly, Parkin plays a minimal role in clearing cardiac mitochondria in POLG mice as cardiac aging is unaffected by cardiac-specific overexpression or global deletion of Parkin (66). Instead, hearts in aged POLG mice have elevated levels of the mitophagy receptor BNIP3 coupled with enhanced mitochondrial biogenesis, indicating enhanced baseline mitochondrial turnover (66). The fact that NIX/BNIP3 double knockout mice accumulate dysfunctional mitochondria in the heart at an accelerated rate with age compared to wild type mice confirms that these mitophagy receptors play a key role in baseline mitochondrial maintenance (68). Furthermore, Rana et al. recently demonstrated that promoting Drp1-mediated mitochondrial fission in midlife leads to increased mitophagy and rejuvenated mitochondria in flies. This leads to improved health span and delays the onset of pathology linked to aging (69). Together, these findings support the notion that reduced mitophagy might be a significant underlying factor in the accumulation of dysfunctional mitochondria in aged organisms contributing to their health decline and mortality. Also, the mitophagy pathway may represent a therapeutic target to counteract aging.

## Age-Related Reduction in Autophagy and Mitophagy

Although autophagy is clearly diminished with age in tissues, including the heart (5, 9–12), exactly why cardiac autophagy is reduced during aging is still unclear. Most of our current knowledge comes from studies in cell lines or other tissues. Oxidative stress can inhibit autophagy by promoting oxidation of the autophagy enzymes involved in autophagy (70). Under baseline conditions when autophagy is not activated, LC3 is covalently bound to inactive Atg3 and Atg7, which protects cysteine residues in their catalytic sites from oxidation. However, the release of LC3 upon activation of autophagy leads to exposure of the cysteines, making them available to direct oxidation during high levels of oxidative stress (70). Moreover, Parkin is also prone to oxidation of its cysteine residues which affects its E3 ubiquitin ligase activity and promotes its misfolding and aggregation (71, 72). Also, both PINK1 and Parkin can be S-nitrosylated which leads to attenuated mitophagy (73, 74). As cardiac aging is characterized by increased oxidative stress (75, 76), it is possible that this directly contributes to reduced autophagosome formation and impaired Parkin-mediated mitophagy in aged myocytes.

Low levels of chronic inflammation has also been linked to age-related diseases (77). The NLRP3 inflammasome is a cytosolic protein complex that initiates activation of inflammatory responses by inducing cell death and triggering the release of proinflammatory cytokines (77). Deregulation of the NLRP3 inflammasome has been linked to inhibition of autophagy and aging. NLRP3-deficient mice have improved health span and attenuated age-related functional decline, including reduced bone loss, improved memory and cognitive performance, and motor performance (78). Recently, it was reported that aged NLRP3-deficient mice have reduced cardiac hypertrophy and fibrosis and increased life spans compared to wild type mice (14). This study linked the NLRP3-deficiency in aged mice to reduced mTOR suppression resulting in increased autophagic activity (14).

Moreover, it is also likely that proteins involved in regulating autophagy are altered with age. For instance, Rubicon is a negative regulator of Beclin1 and it was recently reported that Rubicon expression increases in worm, fly and mouse tissues with age (5). Rubicon knockdown ameliorates age-dependent phenotypes and extends life span in both worms and flies, while Rubicon systemic-knockout mice have reduced age-associated phenotypes such as decreased kidney fibrosis (5). This suggests that Rubicon could be one of the factors contributing to the decline in autophagy during aging. However, other regulators might also be altered with age in tissues.

Finally, lysosomes function in the terminal step of autophagy (Figure 1) and lysosomal function is compromised with age (79). For instance, the activity of lysosomal hydrolases responsible for degrading cargo is dependent on the acidic milieu of the lysosome. After fusion with an autophagosome, the lysosome must undergo reacidification to restore the acidic pH and activate the hydrolases. The v-type ATPase is responsible for maintaining the acidic milieu by pumping proton into the lysosomal lumen and studies indicate that the v-ATPase activity and acidification are reduced with age (80). Lysosomal dysfunction has been identified in age-related neurological pathologies, such as Parkinson's and Alzheimer's disease (80). Lysosomal impairment has also been associated with decreased lifespan, while enhancing lysosomal functional capacity can promote longevity (81, 82). In addition, the adult brain contains a pool of neural stem cells (NSCs) that can generate new neurons but the function of NSCs declines with age. Interestingly, there is an age-dependent decrease in lysosome levels in NSCs which results in fewer lysosomes available to fuse with autophagosomes (83). It is currently unclear if lysosomal function is altered in the aged heart.

## Conclusion

In summary, declines in autophagy and mitophagy in tissues clearly play a role in the aging process and contribute to development of age-related diseases. The main questions that remain unanswered include: why are autophagy and mitophagy suppressed with age and can these pathways be restored in the aged heart? Relatively little is still known about the molecular mechanism underlying the decrease in autophagy and mitophagy and whether there are tissue specific differences. Although manipulation of autophagy and mitophagy pathways are protective in pre-clinical models, the level of activity must be carefully monitored as excessive autophagy can lead to excessive degradation of key cellular components. Increased knowledge into how these pathways are regulated as well as altered with age will allow for more specific manipulation. Further understanding will also provide important insights into how future therapies can protect the heart against age-specific functional decline.

## Author Contributions

Both authors contributed to the content of this article and have approved of its submission.

### Conflict of Interest

The authors declare that the research was conducted in the absence of any commercial or financial relationships that could be construed as a potential conflict of interest.
